# Malarial Diseases

**Published:** 1878-05

**Authors:** 


### *MALARIAL DISEASES.*

Professor Tyndall recognizes as septic affections all those types of zymotic disease which are classed under the term *malarial*, by which is meant chills and fever, the agues, and the various fevers having periodical increase and diminution. Professor Huxley takes the same view, and urges the antiseptic treatment. Dr. Hall goes a step further, and establishes the fact that with the septic state a liver complication also exists, and that this latter indication must be met, or a cure is delayed.

He argues that the value of quinine and arsenic in malarial affections lies chiefly in the fact that they are anti-fermentives, and that the injury which they inflict as poisons is out of all proportion to their usefulness.

He combines mild antiseptics with such extractive substances as are found to gently stimulate the liver, and the scientific world admit that the result proves the truthfulness of his theory. The Messrs. Hall & Ruckel, wholesale druggists of New York, have put up his prescription in two neat boxes under one wrapper, and sell it as "Dr. Hall's Complete Cure for Fever and Ague," for fifty cents

